# Acute cardiac complications and subclinical myocardial injuries associated with pheochromocytoma and paraganglioma

**DOI:** 10.1186/s12872-021-02013-6

**Published:** 2021-04-21

**Authors:** Jing Zhou, He Xuan, Yunxiang Miao, Junting Hu, Yunlang Dai

**Affiliations:** 1 Department of Cardiology, The First Affiliated Hospital of Soochow University, 188 Shizi Road, Suzhou City, 215006 People’s Republic of China; 2 Department of Echocardiography, The First Affiliated Hospital of Soochow University, Suzhou City, Jiangsu Province People’s Republic of China

**Keywords:** Pheochromocytoma, Cardiac complication, Myocardial injury, Longitudinal strain

## Abstract

**Background:**

Catecholamine excess arising from pheochromocytomas and paragangliomas (PPGLs) can cause a wide spectrum of cardiac manifestations, including acute cardiac complications (ACCs) and subclinical myocardial injuries (SMIs). In this study, we aimed to conduct a comprehensive analysis of ACCs and SMIs in a large cohort of patients with PPGLs.

**Methods:**

We retrospectively analyzed the clinical data of consecutive patients with PPGLs admitted between January 2013 and July 2020 (n = 189). The prevalence of ACCs and SMIs and characteristics of patients identified with ACCs and SMIs were investigated. Moreover, comparisons were performed between patients with and without ACCs.

**Results:**

Fourteen patients (7.4%) fulfilled the criteria for ACCs, including nine (4.8%) who presented with Takotsubo-like cardiomyopathy, four (2.1%) with heart failure with preserved ejection fraction, and finally one (0.5%) with catecholamine-induced cardiomyopathy. Compared to those without ACCs (n = 175), patients with ACCs had a higher prevalence of epinephrine-producing PPGLs (81.8% vs 33.9%, *P* = 0.006) and were more likely to show invasive behavior (61.5% vs 27.3%, *P* = 0.022) or hemorrhage/necrosis (53.9% vs 17.4%, *P* = 0.005) on histology. The apical sparing pattern (5/7, 71.4%) was the dominant impairment pattern of longitudinal strain (LS) for patients displaying Takotsubo-like cardiomyopathy. In patients without cardiac symptoms, a fairly high proportion (21/77, 27.3%) of patients who underwent screening for troponin and/or natriuretic peptide and/or echocardiography had SMIs.

**Conclusions:**

One in every fourteen PPGL patients presented with ACCs, and in the patients with Takotsubo-like cardiomyopathy, the apical sparing pattern was the primary impairment pattern of LS. Additionally, nearly one-third of patients without symptoms had SMIs. The diagnosis of PPGLs should be considered in patients with acute reversible cardiomyopathy, especially in those exhibiting an apical sparing pattern of LS.

## Background

Pheochromocytomas (PHEOs) and paragangliomas (PGLs) (PPGLs), secreting catecholamines (CAs), are rare tumors and have a wide variety of cardiovascular manifestations and complications, which were reviewed recently [1]. Clinically, the presentations of PPGLs are usually nonspecific, and only a limited proportion of patients display the classic triad of headache, palpitation and sweating, resulting in delays in diagnosis, especially in patients presenting with cardiac symptoms [2, 3]. Acute cardiac complications (ACCs) due to an excess amount of CAs, including epinephrine, norepinephrine, and sometimes dopamine, in patients with PPGLs may be life-threatening; hence, timely diagnosis and surgery can be life-saving [1]. To date, a few retrospective series of PPGLs have been reported from cardiac perspectives, revealing that the prevalence of ACCs varies from 11% to 19.3% [4–8].

ACCs related to PPGLs encompass arrhythmias (bradycardia and tachycardia), Takotsubo-like cardiomyopathy, dilated cardiomyopathy, thromboembolism, acute coronary syndrome, and so on [1]. PPGLs are considered secondary causes of Takotsubo syndrome (TTS) in the European Society of Cardiology (ESC) expert consensus, and dozens of cases have been reported so far [3, 9].

In the current study, we retrospectively reviewed patients diagnosed with PPGLs in our center and screened carefully for patients with ACCs. The prevalence and features of PPGLs and outcomes of PPGL patients with ACCs were assessed; furthermore, comparisons between patients with and without ACCs were conducted to identify the patient features associated with ACCs. Additionally, subclinical myocardial injuries (SMIs) were also investigated to reveal CA-induced cardiac damage that did not cause clinical symptoms.

## Methods

### Study populations

Consecutive patients diagnosed with PPGLs (clinical or histopathological diagnoses) and admitted between January 2013 and July 2020 were included for analysis. Clinical diagnoses were established by classic CA-related paroxysmal complaints (headache, palpitations, and/or profuse sweating) in combination with adrenal tumors with features suggestive of PPGLs on computed tomography (CT) or magnetic resonance imaging (MRI) and elevated plasma CA or metanephrine (MN)/normetanephrine (NMN) levels at least two times the upper limit of normal (ULN) [10]. In total, 189 patients identified with PPGLs were included in the analysis, of whom 185 had their diagnoses confirmed by histopathology.

Meanwhile, we searched electronic medical records for patients diagnosed with acute coronary syndrome (ACS) and/or acute heart failure (AHF), aiming to draw a picture of the prevalence of PPGLs in these patients.

### Clinical assessments

Data on demographic factors, patterns of hypertension, clinical presentations, electrocardiogram (ECG) alterations, biochemical examinations, preoperative plasma CA and MN/NMN levels, echocardiography results, characteristics of tumors, and in-hospital outcomes were recorded. The patterns of hypertension included normal, sustained (just as essential hypertension), paroxysmal (paroxysms of hypertension on a background of normal blood pressure), mixed (paroxysms of hypertension on a background of sustained hypertension) and unknown patterns [2]. Hypertension was based on the diagnosis at admission and preoperative blood pressure readings, as blood pressure might decline after surgery. Two classification approaches were used for the patterns of secretion: (1) nonsecreting, epinephrine predominant (≥ 2 × ULN, including MN), norepinephrine predominant (≥ 2 × ULN, including NMN), and a combination of both; and (2) with and without epinephrine secretion (≥ 2 × ULN, including MN) [4]. Maximal tumor diameters were comprehensively determined by gross pathological specimens and preoperative CT/MRI. The estimated glomerular filtration rate (eGFR) was calculated using the CKD-EPI method [11].

The diagnoses of ACCs were established in the presence of at least one of the following clinical situations before surgery: (1) Takotsubo-like cardiomyopathy diagnosed by the following combination [9]: (I) transient left ventricular dysfunction with wall-motion abnormality extending beyond a single epicardial coronary artery distribution; (II) either absence of significant coronary artery stenosis confirmed by coronary artery angiography (CAG) or coronary computed tomography angiography (CTA), or no risk factors; (III) ECG abnormalities or elevated cardiac biomarkers; and IV) no evidence of viral infection; (2) heart failure with unknown aetiologies, most likely attributed to CA release from PPGLs, requiring hospitalizations or intravenous diuretic therapy; and (3) systemic thromboembolism of presumed cardiac origin, arrhythmias requiring hospitalizations or antiarrhythmic therapy, and any other cardiac event that was believed to be related to PPGLs.

In addition, SMIs were assessed to reveal detectable myocardial insults before surgery in patients without cardiac symptoms. An SMI was defined as any identified abnormality in myocardial injury biomarkers (high-sensitivity cardiac troponin T) or N-terminal pro-B type natriuretic peptide (NT-proBNP) levels or systolic ventricular dysfunction identified using two-dimensional (2D) strain analysis in the absence of overloading or other conditions that could alternatively explain the abnormal findings.

In suitable patients, 2D strain analysis was performed offline by a single experienced examiner (Y.X. Miao) using dedicated software (EchoPAC, Norway). Data on parameters including global longitudinal strain (GLS), impairment patterns of longitudinal strain (LS) (e.g., apical; apical sparing which was defined as segments in midventricular and basal regions with reduced LS, whereas the segment in the apical region had relatively normal LS; segmental; and global) and any other valuable information were documented.

All patient records were anonymized before analysis and the Institutional Review Board (IRB) waived the requirement for informed consent due to the retrospective nature of the study. The study protocol was approved by the IRB of Soochow University and followed the principles of the Helsinki Declaration.

### Statistical analysis

Continuous variables were assessed for normality by the skewness/kurtosis test and expressed as the mean ± standard deviation (SD) or median (25th, 75th percentile), as appropriate. Means were compared using a 2-tailed Student’s t-test or, in the case of non-normal data, the rank sum test. Categorical variables were shown as numbers (percentages), and differences were detected using the Pearson chi-square test or Fisher’s exact test, where appropriate. All analyses were carried out using STATA version 14.0 (Stata Corp, College Station, TX). A two-tailed* P* value less than 0.05 was considered statistically significant.

## Results

As shown in Table 1, a total of 189 patients were included for analysis, among whom the median age was 53 (38, 62) years, and 52.9% (100/189) were female. Surprisingly, PPGLs were incidentally found in 59.3% (112/189) of patients, who either had no symptoms or, in symptomatic cases, the symptoms were not considered to be related to the space-occupying effect of tumors or CA-related effects. Hypertension occurred in 97 (51.3%) subjects, and a sustained pattern (52/97, 53.6%) was dominant over paroxysmal (15/97, 15.5%), mixed (19/97, 19.6%), and unknown (11/97, 11.3%) patterns. With regard to symptoms documented in the medical records, abdominal discomfort (nausea or vomiting), chest pain, dyspnea and classic triad symptoms (headache, palpitation, and/or profuse sweating) appeared in 9.5% (18/189), 3.2% (6/189), 14.8% (28/189) and 8.5% (16/189) of the included subjects, respectively. Troponin (55/189, 29.1%) and NT-proBNP (51/189, 27.0%) levels were tested in only approximately one-quarter of the study subjects. Seventy (37.0%) patients were screened for tumors with CA secretion, as the plasma CA/MN/NMN test was unavailable until January 2018 in our center; however, the urine vanillylmandelic acid test was performed in 103 (54.5%) cases, resulting in 135 (71.4%) patients undergoing at least one of the aforementioned endocrine tests. The majority of patients underwent a routine urine examination (185/189, 97.9%), ECG examination (182/189, 96.3%), blood count examination (187/189, 98.9%), and renal function examination (188/189, 99.5%). Of note, invasive behavior and the presence of hemorrhage/necrosis on pathology reports were described in 29.7% and 20.0% of cases, respectively. Moreover, the proportion of patients who took antihypertensive medicine declined from 38.1% on admission to 12.2% at discharge (*P* < 0.05).

**Table 1:** Clinical profiles of patients diagnosed with PPGLs and comparisons between the ACC group and the non-ACC group

	All (n = 189)	Non-ACC (n = 175)	ACC (n = 14)	*P* value
Male	89 (47.1)	79 (45.1)	10 (71.4)	0.058
Age (years)	53 (38, 62)	54 (38, 62)	48 (38, 52)	0.320
History of hypertension	97 (51.3)	86 (49.1)	11 (78.6)	0.034
Patterns of hypertension (n = 97)				0.266
Sustained	52 (53.6)	49 (57.0)	3 (27.3)	
Paroxysmal	15 (15.5)	13 (15.1)	2 (18.2)	
Mixed	19 (19.6)	15 (17.4)	4 (36.4)	
Unknown	11 (11.3)	9 (10.5)	2 (18.2)	
Symptoms				
Nausea or vomiting	18 (9.5)	11 (6.3)	7 (50.0)	< 0.001
Presence of chest pain	6 (3.2)	4 (2.3)	2 (14.3)	0.065
Presence of dyspnea	28 (14.8)	14 (8.0)	14 (100)	< 0.001
Presence of triad^a^	16 (8.5)	15 (8.6)	1 (7.1)	1.000
ECG changes (n = 182)				
ST-segment changes	19 (10.4)	15 (8.9)	4 (28.6)	0.043
T wave inversion	29 (15.3)	22 (13.1)	7 (50.0)	0.002
Any abnormality^c^	91 (50.0)	77 (45.8)	14 (100)	< 0.001
Blood parameters				
Hemoglobin (g/L) (n = 187)	132.8 ± 20.7	132.5 ± 20.2	136.8 ± 26.4	0.452
Platelet count (× 10^9^/L) (n = 187)	256.1 ± 81.5	251.9 ± 76.6	308. 6 ± 118.5	0.100
White blood cell count (× 10^9^/L) (n = 187)	6.6 (5.5, 8.6)	6.4 (5.4, 8.1)	15.6 (12.1, 18.9)	< 0.001
eGFR (ml/min/1.73m^2^) (n = 188)	108.0 (96.9, 117.8)	108.0 (97.4, 117.8)	106.1 (49.3, 117.3)	0.408
Proteinuria (n = 185)	24 (13.0)	19 (11.1)	5 (35.7)	0.026
Cardiac biomarkers				
Elevation of troponin (n = 55)	19 (34.5)	6 (14.3)	13 (100)	< 0.001
Elevation of NT-proBNP^b^ (n = 51)	38 (74.5)	25 (65.8)	13 (100)	0.023
Patterns of secretion (n = 70)				
Unknown	4 (5.7)	4 (6.8)	0 (0)	
Nonsecreting	12 (17.1)	12 (20.3)	0 (0)	0.044
Epinephrine predominant	5 (7.1)	4 (6.8)	1 (9.1)	
Norepinephrine predominant	25 (35.7)	23 (39.0)	2 (18.2)	
A combination of both	24 (34.3)	16 (27.1)	8 (72.7)	
Patterns of secretion (n = 70)^d^				
Epinephrine secretion	29 (41.4)	20 (33.9)	9 (81.8)	0.006
Tumor characteristics				
Paragangliomas	52 (27.5)	50 (28.6)	2 (14.3)	0.357
Bilateral adrenal tumors (n = 137)	6 (4.4)	5 (4.0)	1 (8.3)	0.429
Right adrenal tumors (n = 137)	70 (51.1)	64 (51.2)	6 (50.0)	0.937
Maximal tumor diameters (cm)	4.5 (3.2, 6)	4.5 (3, 6)	5.5 (4.7, 6.5)	0.053
Invasive behavior at histology (n = 185)	55 (29.7)	47 (27.3)	8 (61.5)	0.022
Hemorrhage/necrosis at histology (n = 185)	37 (20.0)	30 (17.4)	7 (53.9)	0.005
Antihypertensive therapy				
On admission	72 (38.1)	65 (37.1)	7 (50.0)	0.341
At discharge	23 (12.2)^e^	19 (10.9)	4 (28.6)	0.073

Continuous variables are expressed as mean ± standard deviation or median (25th, 75th percentile); categorical variables are presented as number and percentages in parentheses. Missing data varied by variables

*ACCs* acute cardiac complications, *cm* centimetre, *ECG* electrocardiography, *eGFR* estimated glomerular filtration rate, *NT-proBNP* N-terminal pro-B type natriuretic peptide

^a^Defined as presentation of headache, palpitation, and/or profuse sweating

^b^Defined as NT-proBNP on admission equal to or greater than 125 pg/ml

^c^Defined as any abnormal findings on ECG (e.g., arrhythmias, ST-segment changes, T wave changes, high voltage of left ventricle, etc.)

^d^Categorized into two patterns: with and without epinephrine secretion (≥ 2 × ULN, including metanephrine)

^e^*P* < 0.05 compared with admission

### Acute cardiac complications

As depicted in Table 2, fourteen (7.4%) patients suffered ACCs, including nine patients (4.8%) with Takotsubo-like cardiomyopathy, four (2.1%) with heart failure with preserved ejection fraction (HFpEF) and one (0.5%) with catecholamine-induced cardiomyopathy. Two patients displayed recurrent episodes of Takotsubo-like cardiomyopathy, of whom one (case 10, two times) had episodes 2 years apart and one (case 9, three times) had episodes 4 years apart. Predisposing factors were identified in only six (42.9%) cases, consisting of physical activity or defecation in three cases, operation in one case, pregnancy in one case and respiratory tract infection in the remaining case. In addition, labile blood pressure was detected in five patients (35.7%). Regarding the location of the tumors, diagnoses of PGLs were made in only two cases. In the eleven patients who were screened for plasma CA levels, the combination pattern (8/11, 72.7%) was dominant over the patterns of epinephrine predominance (1/11, 9.1%) and norepinephrine predominance (2/11, 18.2%). All thirteen patients with biomarkers for cardiac injuries displayed elevated troponin T (median 437.6 pg/ml) and NT-proBNP levels (median 8946 pg/ml). The mean value of left ventricular ejection fraction (LVEF) in the acute phase was 0.45, with a range of 0.21–0.65; notably, the LVEF reached the normal range by 6–30 days in all nine patients diagnosed with Takotsubo-like cardiomyopathy. Due to familial PHEOs and other endocrine organ involvement, case 4 was diagnosed with multiple endocrine neoplasia type 2 without genetic testing. Case 14, a middle-aged male patient, underwent a repeat echocardiography examination 10 months after surgery, which showed no recovery of LVEF; hence, he was diagnosed with catecholamine-induced cardiomyopathy.

**Table 2:** Characteristics of patients presenting with ACCs

Case	Acute LVEF (%)	Strain analysis	Impairment patterns of LS	Acute global LS	Coronary artery evaluation	Recovery time on imaging	Clinical diagnosis
1	0.38	Yes	Apical ballooning	− 5.5	CTA: (–)	6 days	Takotsubo syndrome
2	0.39	Yes	Apical sparing	− 10.7	CAG: (–)	7 days	Takotsubo syndrome
3	0.45	Yes	Apical sparing	− 4.8	Without risk factors	30 days	Takotsubo syndrome
4	0.21	Yes	Apical sparing	− 6.1	Without risk factors	14 days	Takotsubo syndrome
5	0.62	No	–	–	CAG: (–)	–	HFpEF
6	0.64	Yes	Apical sparing	− 16.8	CTA: (–)	–	HFpEF
7	0.43	No	–	–	CTA: p-RCA 50% stenosis	12 days	Takotsubo syndrome
8	0.61	No	–	–	–	–	HFpEF, ischemic stroke
9	0.36	No	–	–	Without risk factors	11 days	Recurrent Takotsubo syndrome (3 times)
10	0.38	Yes	Segmental	− 21.2	CTA: (–)	11 days	Recurrent Takotsubo syndrome (2 times)
11	0.65	No	–	–	Without risk factors	–	HFpEF
12	0.59	Yes	Apical sparing	− 9.6	Without risk factors	7 days	Takotsubo syndrome
13	0.35	Yes	Apical sparing	− 13.6	CAG: (–)	15 days	Takotsubo syndrome
14	0.28	Yes	Global	–	CAG: (–)	Without recovery in 10 months	Catecholamine-induced cardiomyopathy

Case	Sex/age (years)*	Initial symptoms	Triggers	Labile blood pressure	Location of tumors	Plasma catecholamine (× ULN)		Troponin (pg/ml)	NT-proBNP (pg/ml)	ECG
						E/MN	NE/NMN			
1	50 s/male	Nausea, vomiting, dyspnea, palpitation	Physical activity	No	Left adrenal PHEO	5.9	2.5	1159	> 35,000	Sinus tachycardia, ST depression and T wave inversion in V_4–6_
2	30 s/male	Nausea, vomiting, dyspnea, palpitation, headache	Physical activity	No	Bilateral adrenal PHEO	15.8	4.2	1220	5976	ST depression and T inversion in V_1–6_, prolonged QTc interval
3	10 s/female	Dyspnea	Infection	No	Right adrenal PHEO	Normal	> 12.1	19.3	2985	Sinus tachycardia
4	40 s/male	Dyspnea, palpitation, sweating	No	Yes	Right adrenal PHEO	4.0	Normal	2773	25,492	Sinus tachycardia, nonspecific T wave changes
5	40 s/female	dyspnea	No	No	Left adrenal PHEO	11.2	11.9	77.3	4740	T wave inversion in inferior leads
6	60 s/male	Dyspnea, palpitation, sweating	No	No	Left adrenal PHEO	2.6	57.5	84.3	7976	Diminished R wave in V_2–3_
7	50 s/male	Dyspnea, chest pain	Operation	No	Right adrenal PHEO	–	–	130.2	2250	Premature atrial complex
8	60 s/female	Nausea, vomiting, dyspnea, sweating	Defecation	Yes	Right adrenal PHEO	–	–	437.6	33,914	T wave inversion in inferior leads and V_2–6_
9	20 s/male	Nausea, dyspnea, palpitation, sweating	No	Yes	Left adrenal PHEO	8.4	11.4	663.6	26,508	Sinus tachycardia, second degree AVB
10	70 s/male	Nausea, vomiting, dyspnea	No	No	Right adrenal PHEO	9.4	2.9	2178	> 35,000	Sinus tachycardia, low voltage in limb leads, ST depression in V_3–6_
11	30 s/female	Dyspnea, palpitation, sweating	Pregnancy	Yes	Retroperitoneal PGL	–	–	–	–	T wave inversion in inferior leads and V_3–6_
12	40 s/male	Nausea, vomiting, dyspnea, headache	No	Yes	Right adrenal PHEO	25.1	16.0	121.9	8946	T wave inversion in I, aVL, and V6
13	40 s/male	Nausea, vomiting, dyspnea, chest pain, headache	No	No	Left adrenal PHEO	10.4	7.6	954.2	12,913	ST depression in inferior leads and V_4–6_, ST elevation in aVR
14	50 s/male	Dyspnea	No	No	Pelvic PGL	Normal	> 12.1	72.1	1208	Sinus tachycardia, LBBB

*AVB* atrioventricular block, *CAG* coronary artery angiography, *CTA* coronary computed tomography angiography, *E* epinephrine, *HFpEF* heart failure with preserved ejection fraction, *LBBB* left bundle branch block, *LVEF* left ventricular ejection fraction, *LS* longitudinal strain, *LV* left ventricle, *MN* metanephrine, *NE* norepinephrine, *NMN* normetanephrine, *PGL* paraganglioma, *PHEO* pheochromocytoma, *p-RCA* proximal right coronary artery, *ULN* upper limit of normal. Other abbreviations as shown in Table 1

*Ages expressed in decades to ensure patient anonymity in Tables 2 and 3

In comparison to the non-ACC group, the ACC group shared similar characteristics in sex (male, 71.4% vs 45.1%, *P* = 0.058) and age (median, 48 vs 54, *P* = 0.320). As expected, more patients with ACCs had hypertension (78.6% vs 49.1%, *P* = 0.034); however, the patterns of hypertension did not differ between the two groups (*P* = 0.266). Surprisingly, the proportions of subjects who displayed classic triad symptoms (7.1% vs 8.6%, *P* = 1.000) and chest pain (14.3% vs 2.3%, *P* = 0.065) were similar between the two groups and quite low in both; nevertheless, significantly more patients showed gastrointestinal symptoms (50.0% vs 6.3%, *P* < 0.001) and dyspnea (100% vs 8.0%, *P* < 0.001) in the ACC group. As expected, with regard to ECG changes, ST-segment changes (28.6% vs 8.9%, *P* = 0.043), T wave inversions (50.0% vs 13.1%, *P* = 0.002), and any abnormality (100% vs 45.8%, *P* < 0.001) were more prevalent in patients with ACCs than in those without ACCs. In terms of laboratory findings, proteinuria was more likely to be found in the ACC group (35.7% vs 11.1%, *P* = 0.026); likewise, the ACC group had a higher median level of white blood cells (median, 15.6 vs 6.4, *P* < 0.001). Regarding patterns of secretion, a combination pattern was dominant in patients with ACCs (*P* = 0.044); moreover, there was a higher prevalence of epinephrine-producing PPGLs (81.8% vs 33.9%, *P* = 0.006) among the ACC group. Overall, the proportions of paragangliomas, bilateral adrenal tumors, and right adrenal tumors were similar between the two groups; however, patients with ACCs exhibited a trend towards having larger maximal tumor diameters, but the difference did not reach statistical significance (median, 5.5 vs 4.5, *P* = 0.053). Interestingly, more patients in the ACC group showed invasive behavior (61.5% vs 27.3%, *P* = 0.022) and hemorrhage/necrosis (53.9% vs 17.4%, *P* = 0.005) in pathology reports than patients in the non-ACC group. Finally, with respect to the proportion of patients who received antihypertensive therapy, two groups did neither differ on admission nor at discharge.

During the study period, 9432 patients diagnosed with ACS and/or AHF were identified from the electronic search, resulting in a prevalence of 0.15% for PPGL-induced ACCs in these patients.

### Strain analysis

**Fig. 1:**
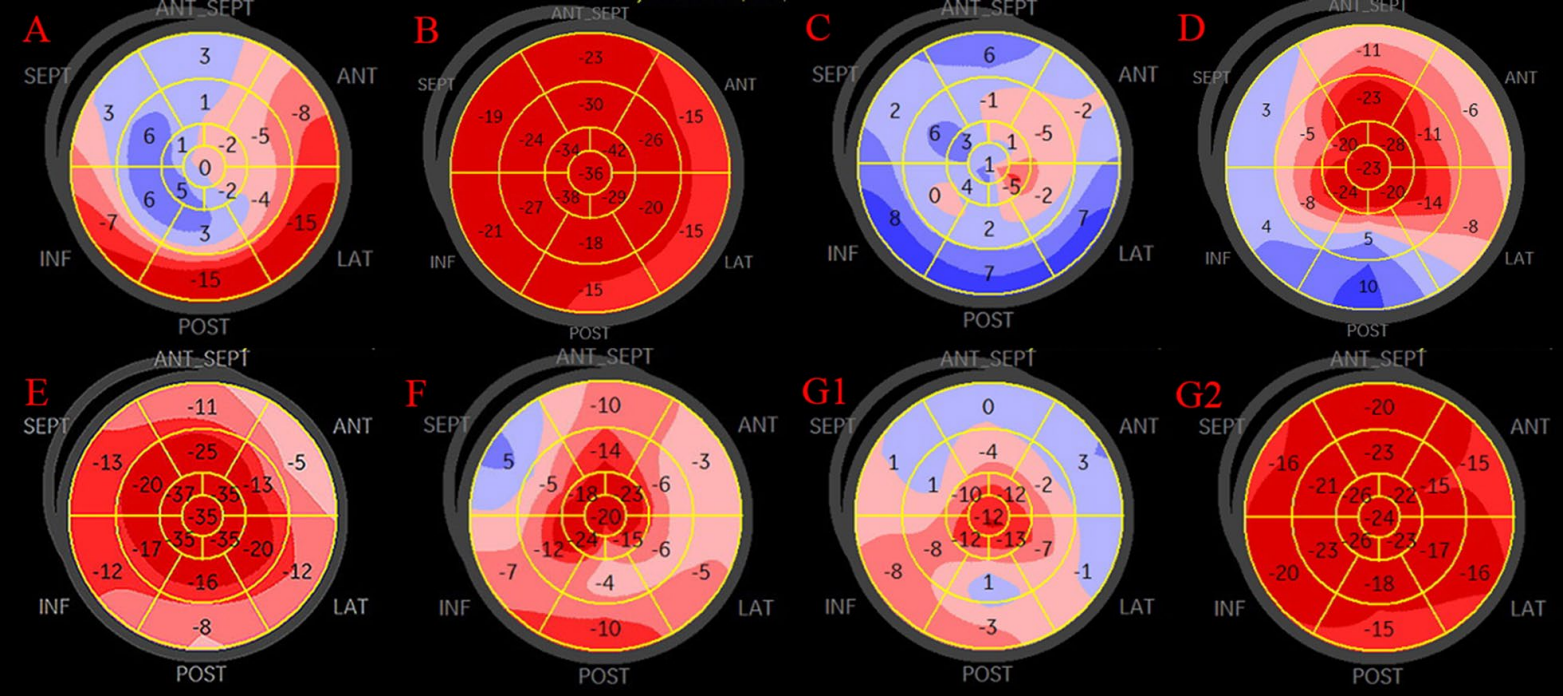
Representative impairment patterns of longitudinal strain for each subgroup. **a** The pattern of apical ballooning in case 1. **b** The pattern of segmental dysfunction in case 10. **c** The pattern of global dysfunction in case 14. **d**–**f** The patterns of apical sparing in cases 2, 6, and 12. **g1, 2** The apical sparing pattern during the acute phase and a nearly full recovery 14 days later in case 4

As shown in Table 2, 2D strain analysis was carried out in 9 (64.3%) out of 14 patients with ACCs, and technical reasons accounted for failure to perform the analysis in the remaining 5 cases. Strikingly, 6 (66.7%) patients displayed a pattern of relative apical sparing of LS; meanwhile, of the remaining 3 patients, one (case 1) showed a pattern of apical ballooning, one (case 10) displayed a pattern of segmental dysfunction, and the last patient (case 14) showed a pattern of global dysfunction. Of the 7 patients with Takotsubo-like cardiomyopathy who underwent strain analysis, one patient (case 1) (14.3%) exhibited a typical apical ballooning pattern; however, 5 (71.4%) (cases 2, 3, 4, 12, 13) patients showed a pattern of relative apical sparing of LS. Visual examples of the patterns of ‘apical ballooning’, ‘apical sparing’, ‘global’ and ‘segmental’ of LS are depicted in Fig. 1a–g. In addition, the mean value of GLS was − 11.0 ± 5.8, with a range of − 5.5 to − 21.2.

### Subclinical myocardial injuries

The prevalence of SMIs can be potentially underestimated owing to unthorough investigations or be overestimated because the decision to perform examinations was made at the discretion of the physicians in this study. Among 175 patients without ACCs, only 60 (34.3%) patients underwent echocardiography examinations, 42 (24.0%) received troponin tests and 38 (21.7%) underwent NT-proBNP tests; furthermore, 77 (44.0%) patients received at least one of the aforementioned three examinations, among whom 21 (27.3%) patients experienced SMIs, including elevated NT-proBNP levels in 15, elevated troponin in 4 and abnormal findings on echocardiography examinations in 10 patients (shown in Table 3). Additionally, among the above 77 patients, 40 exhibited any abnormality on ECG, including 7 (17.5%) who fulfilled the criteria for SMIs.

**Table 3:** Characteristics of patients with SMIs

Case	Sex/age (years)	Location of tumors	Blood catecholamine (× ULN)		Troponin(pg/ml)	NT-proBNP(pg/ml)	ECG	Coronary artery evaluation	Echocardiography	Impairment patterns on strain analysis*
			E/MN	NE/NMN						
1	50 s/male	Retroperitoneal PGL	Normal	15.3	Normal	Normal	Normal	CTA: p-RCA mild stenosis	Enlarged LA and LV, LVH	Anterior and lateral segments
2	30 s/female	Right adrenal PHEO	Normal	33.1	–	–	ST depression and T wave inversion in inferior leads and V_4–6_	Without risk factors	LVH	Apical sparing
3	30 s/male	Retroperitoneal PGL	–	–	21.4	216.4	T wave inversion in inferior leads and V_4–6_	Without risk factors	LVH	–
4	50 s/male	Retroperitoneal PGL	Normal	12.2	337.4	137.2	Normal	CTA: (–)	Normal	Normal
5	40 s/female	Left adrenal PHEO	–	–	Normal	350	Normal	CTA: p-LAD 20% stenosis	Enlarged LA	–
6	50 s/female	Left adrenal PHEO	9.1	2.7	Normal	366.6	T wave flat in inferior leads and V_4–6_	Without risk factors	Normal	Apical sparing
7	30 s/male	Left adrenal PHEO	Normal	> 12.1	Normal	164.2	LVH	Without risk factors	Enlarged LA, LVH	anterior and lateral segments
8	60 s/male	Retroperitoneal PGL	–	–	21	2140	Normal	CAG: p-LAD 40% stenosis	–	–
9	70 s/male	Left adrenal PHEO	4.9	4.8	–	–	Normal	CTA: (–)	Normal	Basal inferior segment
10	50 s/female	Right adrenal PHEO	–	–	Normal	153	–	Without risk factors	Normal	Anterior and lateral segments
11	50 s/female	Right adrenal PHEO	38.9	12.1	Normal	778.4	ST depression in V_4–6_ and biphasic T wave in inferior leads and V_3–6_	Without risk factors	LVH	–
12	60 s/female	Left adrenal PHEO	Normal	5.2	–	–	Incomplete RBBB	–	Enlarged LA, LVH	Anterior and lateral segments
13	30 s/male	Left adrenal PHEO	Normal	8.4	Normal	303.8	normal	Without risk factors	–	–
14	30 s/female	Left adrenal PHEO	7.6	9.9	Normal	1008	Shortened PR interval	Without risk factors	Normal	Anterior and lateral segments
15	60 s/male	Right adrenal PHEO	2.8	7.4	–	–	–	CTA: p-LCX mild stenosis	Segmental dysfunction, enlarged LA	Anterior and lateral segments
16	50 s/female	Right adrenal PHEO	4.0	1.9	Normal	322.6	Normal	Without risk factors	–	–
17	50 s/female	Right adrenal PHEO	Normal	Normal	–	–	Normal	Without risk factors	Pulmonary hypertension	Anterior segment
18	30 s/female	Right adrenal PHEO	4.0	4.0	Normal	408.5	Normal	Without risk factors	–	–
19	20 s/male	Right adrenal PHEO	Normal	8.5	Normal	337.7	Normal	Without risk factors	–	–
20	50 s/female	Right adrenal PHEO	Normal	Normal	48.39	215.8	Normal	–	–	–
21	40 s/female	Left adrenal PHEO	–	–	Normal	770	Normal	Without risk factors	–	–

LA left atrium, LV left ventricle, LVH left ventricular hypertrophy, p-LAD proximal left anterior descending coronary artery, p-LCX proximal left circumflex coronary artery, RBBB right bundle branch block, SMIs subclinical myocardial injuries. Other abbreviations as shown in Tables 1 and 2

*Not all patients with echocardiography examinations were suitable for two-dimension strain analysis

## Discussion

In this retrospective study with a large sample size (189 cases), we demonstrated a relatively high prevalence (7.4%) of ACCs owing to CA excess in patients with PPGLs. Moreover, 9 out of 14 patients with ACCs displayed Takotsubo-like cardiomyopathy, and 2D strain analysis further revealed that the predominant impairment pattern was the apical sparing pattern (5/7), which was dominant over the typical apical ballooning pattern (1/7) seen in TTS. In fact, we believed that some, if not all, patients diagnosed with HFpEF had gone through the clinical course of Takotsubo-like cardiomyopathy, since strain analysis showed impaired strain in case 6. Of note, a fairly high incidence (27.3%) of SMIs was observed in patients who underwent cardiac biomarker and/or echocardiography examinations, suggesting the universality of CA-related cardiac damage.

In this study, the majority (59.3%) of PPGLs were found incidentally based on abdominal ultrasonogram or CT/MRI scans rather than PPGL-related symptoms. The main discovery method is consistent with that in recent cohort studies, implying changes in PPGL recognition due to the widespread use of cross-sectional imaging; hence, the relatively low prevalence of hypertension and symptoms might be explained by the fact that most PPGLs have been incidentally found in recent years [12, 13]. Nausea or vomiting occurred in half of the patients with ACCs, which should be partly, if not totally, ascribed to excessive CA secretion [13]. These data were surprising to us given less than ten percent of patients showed classical triad symptoms; this rate was lower than the prevalence (17%) reported by a recent study [13] and might be underestimated owing to the retrospective nature of the study. However, a low prevalence (4%) of the classical triad in patients with PPGL-induced reversible cardiomyopathy was also reported by a recent review, which could help remind clinicians to consider PPGLs in the context of reversible cardiomyopathy with unclear aetiology and not to rely on the classical triad as a diagnostic threshold [3].

Proteinuria was more prevalent in patients with ACCs than in non-ACC controls, implying more severe kidney injury caused by CAs or cytokines released from PPGLs [14]. Notably, epinephrine secretion was present in most patients (81.8%) with ACCs, which was consistent with previous studies [4], indicating that the episodical release of epinephrine caused sudden cardiac decompensation as opposed to the persistent storage and release of norepinephrine [15, 16]. A trend towards a larger tumor diameter in the ACC group than in the non-ACC group (*P* = 0.053) was observed, but inconsistent correlations between tumor size and ACCs have been reported in prior studies [5–7]. The presence of hemorrhage/necrosis on histology was more often seen in subjects with ACCs than in those without, suggesting a possible role as triggers played by hemorrhage/necrosis [2, 17]. In agreement with the recognition that all PPGLs have a metastatic risk, we reported a fairly high rate of invasive behavior on pathology reports (29.7%). Additionally, invasive behavior seemed to be associated with ACCs; however, the result should be considered exploratory owing to the lack of standardized interpretation of pathology results and well-accepted histological features indicating metastasis for PPGLs [18].

The prevalence (4.8%) of Takotsubo-like cardiomyopathy in our cohort was comparable with the prevalence of 1.4 ~ 5.6% reported in previous studies [4–8]. The apical sparing pattern (5/7), indicating the impairment of segments in the mid-ventricular and basal regions, was the dominant impairment pattern of LS, whereas prior reviews revealed a predominance (48%) of the classical apical ballooning pattern on routine echocardiography examinations [3, 4]. The different impaired patterns between PPGL-induced TTS and classic TTS may hint that distinct pathogenetic mechanisms exist [19, 20]. Recurrent TTS associated with PPGLs was recently reported and systematically reviewed by our team [21]; of note, two patients in this cohort who experienced recurrent episodes of TTS were diagnosed with myocarditis 2–4 years before PPGLs were identified, highlighting the value of screening for PPGLs among these patients. Although, as revealed in this study, the prevalence of PPGLs was extremely low (0.15%) in the general population presenting with ACS and/or AHF, clinicians should consider such tumors when the patients exhibit the following features: no risk factors for heart failure, labile blood pressure, presence of a retroperitoneal tumor, and Takotsubo-like cardiomyopathy (especially displaying an apical sparing pattern of LS).

Unexpectedly, a quite high prevalence (27.3%) of SMIs in patients with PPGLs was observed in our study, implying that cardiac injuries without clinical manifestations are common in the presence of excessive CA release. Abnormalities on ECG examinations, including sinus tachycardia, atrial fibrillation, premature complex, and nonspecific ST-T changes, were not deemed SMIs because they were nonspecific for PPGL-related cardiac damage; hence, the occurrence rate of SMIs might be underestimated.

### Strengthens and limitations of this study

This study is the first study to investigate PPGL-induced ACCs and SMIs in an East-Asian population using a large sample size. This is the first study to characterize echocardiography abnormalities on strain analysis and demonstrate that the primary impairment pattern is the apical sparing pattern. In addition, the high prevalence, which is possibly underestimated, of SMIs observed in this study has not been reported in previous cohort studies. However, limitations exist, including the following: first, we were not able to perform comprehensive assessments of the cardiac injuries associated with exceedingly high levels of CAs because of the retrospective nature of the study; second, due to lack of genetic screening, the diagnoses made in this cohort failed to meet the requirements recommended by current guidelines [10]; third, the low number of events made it impossible to conduct regression analyses to reveal the independent factors associated with ACCs; and finally, these results should be generalized with caution owing to center-specific referral bias.

## Conclusion

Collectively, this retrospective study revealed that one in every fourteen PPGL patients presented with ACCs. The main impairment pattern in patients with TTS on strain analysis was the apical sparing pattern, which is a distinct result from previous studies. Moreover, nearly one-third of patients without symptoms had SMIs. Clinical clues suggestive of PPGLs should not solely include adrenergic symptoms, particularly the classic triad, due to their extremely low prevalence. Clinicians should consider PPGLs in patients with acute reversible cardiomyopathy, especially in those displaying an apical sparing pattern of LS.

## Data Availability

The datasets used and/or analyzed during the current study are not publicly available due to the protection of individual privacy, but are available from the corresponding author on reasonable request.

## Abbreviations

2D: Two-dimensional
ACCs: Acute cardiac complications
ACS: Acute coronary syndrome
AHF: Acute heart failure
CAG: Coronary artery angiography
CAs: Catecholamines
CT: Computed tomography
CTA: Coronary computed tomography angiography
ECG: Electrocardiography
ESC: European Society of Cardiology
eGFR: Estimated glomerular filtration rate
GLS: Global longitudinal strain
HFpEF: Heart failure with preserved ejection fraction
IRB: Institutional Review Boards
LVEF: Left ventricular ejection fraction
LS: Longitudinal strain
MN: Metanephrine
MRI: Magnetic resonance imaging
NMN: Normetanephrine
NT-proBNP: N-terminal pro-B type natriuretic peptide
PGLs: Paragangliomas
PHEOs: Pheochromocytomas
PPGLs: Pheochromocytomas and paragangliomas
SD: Standard deviation
SMIs: Subclinical myocardial injuries
TTS: Takotsubo syndrome
ULN: Upper limit of normal
